# Towards a Green Hydrate Inhibitor: Imaging Antifreeze Proteins on Clathrates

**DOI:** 10.1371/journal.pone.0008953

**Published:** 2010-02-11

**Authors:** Raimond Gordienko, Hiroshi Ohno, Vinay K. Singh, Zongchao Jia, John A. Ripmeester, Virginia K. Walker

**Affiliations:** 1 Department of Biology, Queen's University, Kingston, Ontario, Canada; 2 Department of Biochemistry, Queen's University, Kingston, Ontario, Canada; 3 Materials Structure and Function Group, National Research Council Canada, Ottawa, Ontario, Canada; University of Southampton, United Kingdom

## Abstract

The formation of hydrate plugs in oil and gas pipelines is a serious industrial problem and recently there has been an increased interest in the use of alternative hydrate inhibitors as substitutes for thermodynamic inhibitors like methanol. We show here that antifreeze proteins (AFPs) possess the ability to modify structure II (sII) tetrahydrofuran (THF) hydrate crystal morphologies by adhering to the hydrate surface and inhibiting growth in a similar fashion to the kinetic inhibitor poly-N-vinylpyrrolidone (PVP). The effects of AFPs on the formation and growth rate of high-pressure sII gas mix hydrate demonstrated that AFPs are superior hydrate inhibitors compared to PVP. These results indicate that AFPs may be suitable for the study of new inhibitor systems and represent an important step towards the development of biologically-based hydrate inhibitors.

## Introduction

Gas hydrates, or clathrates, are ice-like compounds that form when hydrocarbon-based guest molecules are trapped in hydrogen-bonded water cages that form under high pressures and low temperatures [1]. Natural gas hydrates most commonly exist as one of two structures. Small guest molecules such as methane tend to form structure I (sI) hydrates while larger guests like propane form structure II (sII) hydrates [2]. In the laboratory, gas hydrates are conveniently modeled using tetrahydrofuran (THF) which is enclathrated at atmospheric pressures [3]. THF hydrate forms cubic sII clathrates, similar to the hydrates that form in pipelines during oil and gas production [4].

Recently, the petroleum industry has been moving into deeper waters which present prime conditions for hydrate growth. Hydrate plugs impede oil and gas flow, resulting in equipment damage as well as hazardous working conditions that can even result in blowouts [5]. Thermodynamic inhibitors such as methanol are one of the most common practical means of controlling hydrate formation [6]. However, as a result of the high costs, flammability and environmental toxicity associated with such inhibitors, there has been a shift towards the less toxic and sometimes cheaper alternative kinetic hydrate inhibitors, which delay nucleation and interfere with crystal growth, as well as antiagglomerants, which act to prevent hydrates from aggregating into larger masses [7], [8].

These concerns have prompted us to investigate the potential inhibitory effects of antifreeze proteins (AFPs) on hydrates. AFPs are a diverse class of proteins that were first identified in fish during the 1950s and have since been found in cold-adapted bacteria, plants and insects [9]–[13]. Despite differences in structure, they have the common ability to adsorb to ice using specific ice-binding faces. AFPs lower the freezing point of water as a result of increased local curvature of growing ice around the adsorbed protein, resulting in a difference between the freezing and melting points, a phenomenon known as thermal hysteresis (TH) [13].

We have previously proposed AFPs as alternative hydrate inhibitors [14]–[15]. Here we visualize fluorescently-tagged AFPs and characterize the effects of these proteins on THF hydrate crystals. We also determine the inhibitory effects of these AFPs on the gas consumption and growth rates of sII natural gas hydrate as part of our efforts towards the development of alternative, biologically-based hydrate inhibitors.

## Results

### Bioreactor Yields

**Figure 1:**
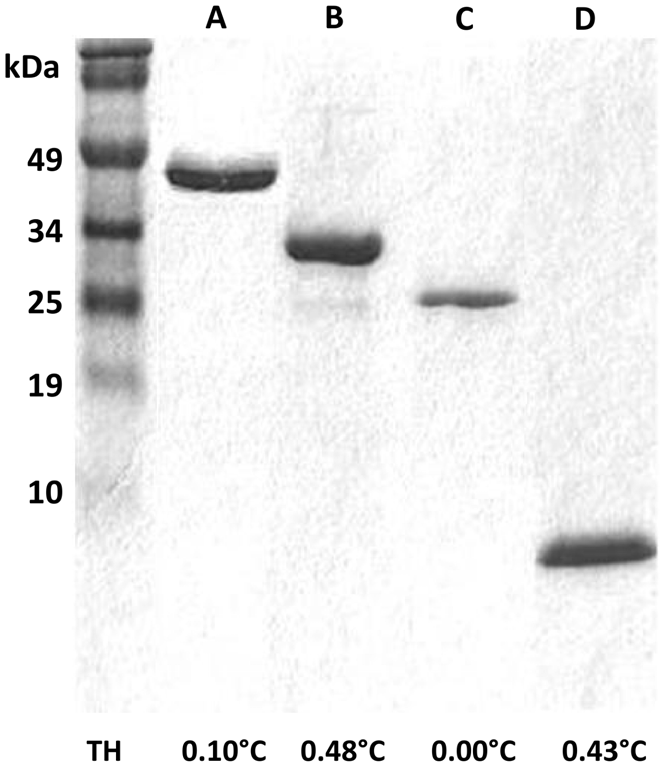
Purification of recombinant proteins. Typical 12% SDS-PAGE analysis of recombinant His-tagged bioreactor-produced proteins including LpAFP-GFP (A), Type III AFP-GFP (B), GFP (C) and Type III AFP (D), purified using Co^2+^-agarose affinity chromatography. Average TH values are shown below.

The AFP cloned from the perennial grass, *Lolium perenne* (Lp), has a low TH while the ocean pout fish Type III AFP is more active [9], [11]. Sequences encoding these AFPs, with or without a green fluorescent protein (GFP) label, were expressed in *E. coli* grown in a bioreactor. Yields were comparable for all recombinant proteins, although optimization of AFP production depended on a variety of conditions including total volume of growth media, level of dissolved oxygen (DO) or OD_600_ at isopropyl β-D-1-thiogalactopyranoside (IPTG) induction as well as temperature during the induction period. Although most of these parameters were kept as constant as possible, it was generally found that lower induction temperatures (≤20°C), yielded the highest amounts of protein. Final purified yields for reactor volumes of 3.5 to 4 L ranged from 180 to 260 mg. Purified recombinant proteins were seen as single bands on a 12% SDS-PAGE gel (Fig. 1). TH values for the AFPs were in the expected ranges at 0.10 (±8.5×10^−3^)°C for LpAFP-GFP, 0.48 (±2.8×10^−2^)°C for Type III AFP-GFP and 0.43 (±2.8×10^−2^)°C for Type III AFP. Control GFP showed no TH. All proteins were tested at ∼4 mg/ml.

### Recombinant GFP-Labeled Proteins and Polycrystalline THF Hydrate

**Figure 2:**
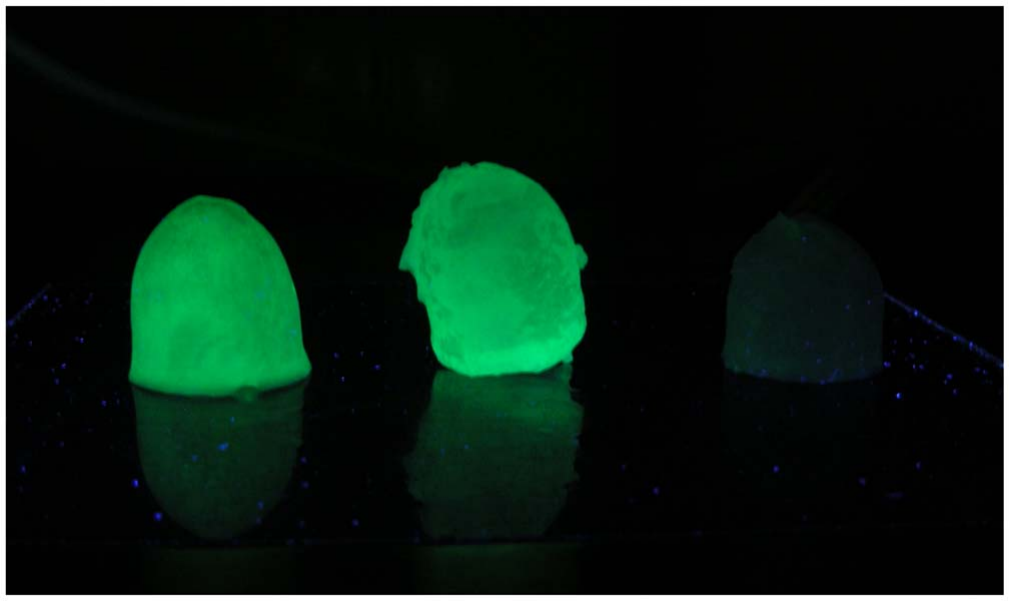
Adsorption of AFPs on THF hydrate. Representative THF hydrate polycrystals fluoresce green under UV light after being grown in solutions containing Type III AFP-GFP (left) and LpAFP-GFP (center). THF hydrate crystals grown in GFP control solutions (right) displayed no fluorescence. Sample diameters were 3–3.5 cm.

**Figure 3 pone-0008953-g003:**
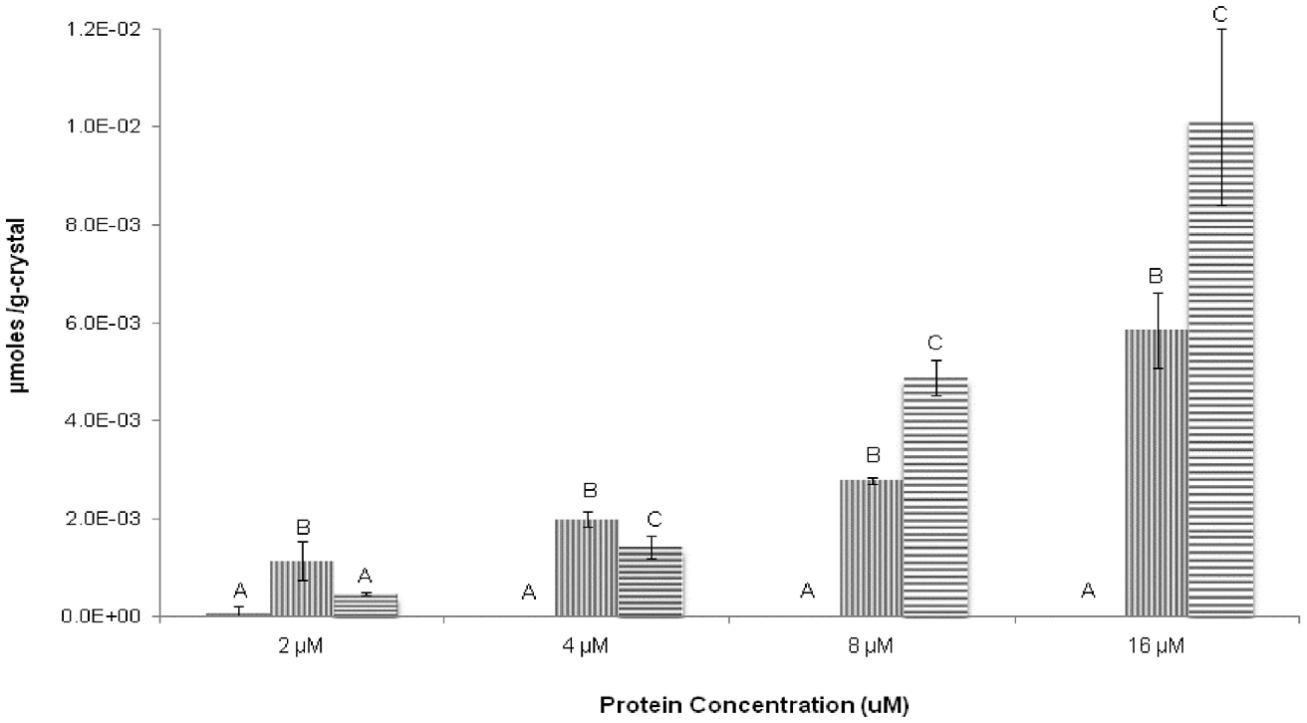
Levels of AFP adsorbed into THF hydrate. Differences in the average µmoles of protein adsorbed per gram of THF hydrate crystal (µmoles/g-crystal) between LpAFP-GFP (horizontal lines), Type III AFP-GFP (vertical lines) and GFP (solid) are plotted as a function of protein concentration (µM). Bars indicate standard deviation. Statistical significance between each data group is indicated by letters A–C, where identical letters indicate no statistical difference.

Polycrystalline THF hydrate crystals grown in the presence of GFP-tagged AFPs were obviously fluorescent green under UV illumination (Fig. 2). Conversely, when hydrate was grown in the presence of recombinant GFP alone, the hydrates were uniformly dark. In an effort to quantify the adsorption, the hydrates were melted and assayed for adsorbed protein (µmoles of recombinant protein per gram of crystal, Fig. 3). For both purified LpAFP-GFP and Type III AFP-GFP fusion proteins, there was a linear correlation between the amount of protein adsorbed into the growing THF hydrate and the concentration of the protein in the THF solution. At lower concentrations (2 and 4 µM) more Type III AFP-GFP appeared to bind than LpAFP-GFP. At higher concentrations (8 and 16 µM) more LpAFP-GFP adsorbed, to an average of 42% more, than Type III AFP. All differences between the two AFP-GFPs were statistically significant. In contrast, no adsorption of GFP was detected in the crystals at any concentration, with the exception of 2 µM, where an average of 7.9×10^−5^ µmoles/g-crystal was detected. At all other concentrations, differences in the amounts of GFP bound compared to the two AFP-GFPs were statistically significant.

### AFPs and Single THF Hydrate Crystals

**Figure 4:**
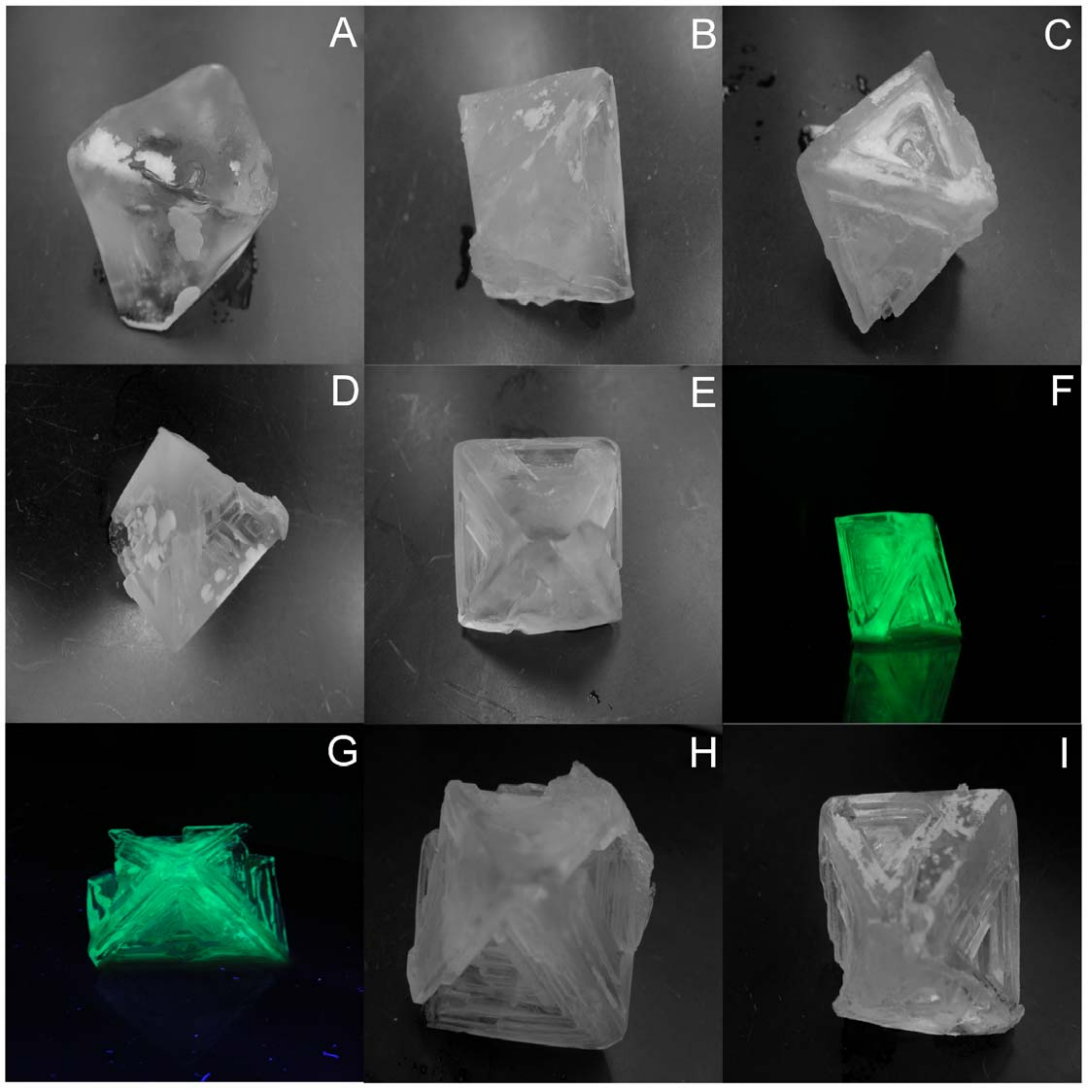
Single THF hydrate crystals. Single crystals grown in solutions of THF/water (A) and 200 µg/ml GFP (B) show no major changes in morphology. Crystals grown in solutions containing 200 µg/ml (30 µM) Type III AFP-GFP (C), 200 µg/ml (4.9 µM) LpAFP-GFP (D) and 15 µg/ml (2.2 µM) Type III AFP (E) show skeletal growth. Crystals with adsorbed Type III AFP-GFP (100 µg/ml; 3.1 µM) (F) and LpAFP-GFP (100 µg/ml; 2.4 µM) (G) show skeletal growth and fluoresce under UV light. Skeletal crystals were grown in 200 µg/ml (20 µM) PVP (H). Crystals (A–H) were grown at 3°C. Control crystals grown in THF/water at high driving force (0°C) show skeletal growth (I). All experiments were done in triplicate and have 4.5–5 cm crystal diameters.

Since the fusion proteins adsorbed to polycrystalline THF hydrate, single THF hydrate crystals were slowly grown at low supercooling (3°C) to observe if the presence of the proteins could change crystal morphology. With no proteins, or in the presence of low to moderate concentrations of GFP (Fig. 4A and B), crystals exhibited a cubic octahedral shape that is characteristic of THF hydrate at these conditions [16]. The {111} planes were clearly defined and there were no apparent modifications to crystal shape.

In striking contrast, when single crystals were grown under the same conditions, but in the presence of recombinant AFPs, they exhibited hopper-like or skeletal morphologies (Fig. 4C–G), indicating interference in normal crystal growth habit [17]. At all but the lowest concentration of LpAFP-GFP, depressions were observed on the interior {111} faces. Similar morphologies were seen in crystals grown in solutions of all but the lowest concentration (15 µg/ml, or 0.472 µM) of Type III AFP-GFP. Interestingly, only the non-GFP tagged Type III AFP displayed clear skeletal shaping at the lowest concentration (15 µg/ml, or 2.2 µM). It was not possible to test this protein at higher concentrations since it precipitated easily in the THF solution. As indicated, recombinant GFP did not appear to alter crystal morphology until tested at very high concentrations (100 and 200 µg/ml, or 4 and 8 µM) and even then showed only one to two shallow face depressions. When the crystals grown in AFP-GFP solutions were observed under UV light, it appeared that the protein had adsorbed uniformly throughout the entire cubic crystal and at every face (Fig. 4F and G). Consistent with the morphological observations, there was no evidence of GFP adsorption under UV light at the low or moderate concentrations used.

In order to compare the effect of a known sII inhibitor on the growth habit of single THF hydrate crystals, crystals were grown in solutions containing poly-N-vinylpyrrolidone (PVP). In all but the lowest concentration of PVP, single crystals were hopper-like and similar to those grown in the presence of AFPs, with the depressions on the {111} faces becoming more pronounced as the concentration of PVP increased (Fig. 4H). It should be noted that all these crystals were grown slowly at temperatures just below the THF hydrate crystallization point. When single crystals were grown at higher driving force, in supercooled conditions at 0°C (Fig. 4I) or at −1.5°C, they exhibited a skeletal morphology like crystals grown in AFPs and PVP.

### AFPs and Gas Hydrates

**Figure 5:**
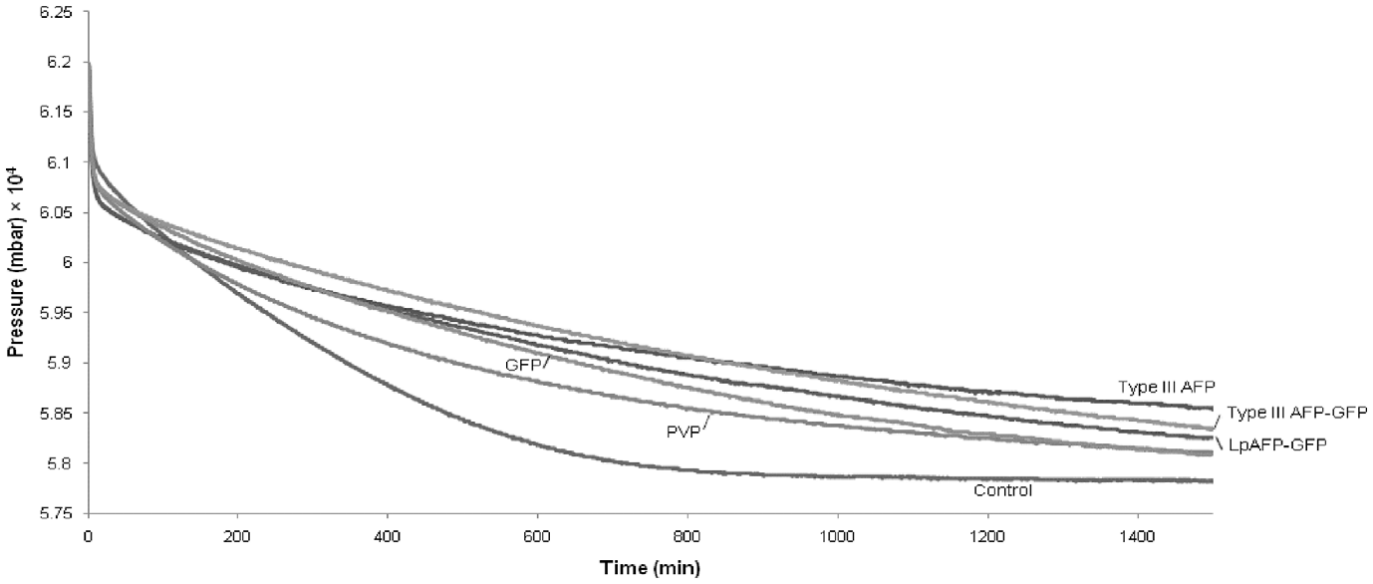
Pressure summary of sII methane/ethane/propane gas hydrate with 0.1 mM additives. Pressure trends plotted against time for Type III AFP, Type III AFP-GFP, LpAFP-GFP, GFP, PVP and control water samples. Absolute pressure drops are proportional to the quantity of moles of gas consumed. Experiments were done in duplicate.

Gas hydrates were formed under high driving force using a natural gas (methane/ethane/propane) mixture so as to produce sII hydrate (Fig. 5). Both average moles of gas consumed (nGas) and average consumption rates followed identical patterns. Because the control samples, without additives, stopped growing at ∼25 h (1 500 min), this time was selected as a cutoff point to compare gas consumption and growth rates. On average, the highest level of inhibition was seen in the presence of Type III AFP (with no GFP fusion protein). In the presence of this protein, hydrate formation consumed 6.0 mmol of gas and showed a decrease in growth rate by 18% (a mean of 4.0×10^−6^ nGas/min) compared to control samples which consumed the highest amount of gas at 7.4 mmol and showed the fastest growth rates (4.9×10^−6^ nGas/min). LpAFP-GFP and Type III AFP-GFP demonstrated modestly lower gas consumption (6.6 mmol<gas consumption<6.4 mmol) and decreased growth rates of 10 and 13%, respectively (4.4×10^−6^ nGas/min<growth rate<4.3×10^−6^ nGas/min). In contrast, the GFP and PVP samples showed the highest levels of gas consumption, except for controls, equally at 6.9 mmol, with a fast growth rate (4.6×10^−6^ nGas/min for both), a 7.1% decrease, compared to the controls.

## Discussion

The observation that the polycrystalline THF hydrates were strikingly fluorescent after being grown in the presence of recombinant AFP-GFPs (Fig. 2) is irrefutable evidence that these proteins adsorb to sII hydrates. Further substantiation is provided by the morphological changes on single hydrate crystals mediated by these same proteins (Fig. 4). This might not have been predicted *a priori* because although hydrates have an ice-like appearance, their structure is markedly different. Under common conditions, water freezes into hexagonal ice (I_h_), taking the form of a hexagonal prism with two basal faces and six rectangular prism faces [18]. Moderately active AFPs such as Type III AFP (and Type III AFP-GFP) have been shown to bind to the ice basal planes, with the low activity LpAFP binding to the prism planes [19], [20]. Thus, evolutionary forces have shaped these AFPs so that they fit securely to these ice surfaces making it more energetically expensive for water molecules to join the growing ice lattice. In contrast, sII hydrates are octahedral with a symmetrical cubic structure and therefore, although similarly flat to ice, present geometrically-distinct, but uniform surfaces for AFP adsorption.

There was no preference by AFPs for any of the 8 identical {111} hydrate faces (Fig. 4F and G), and indeed the resulting hopper-like crystals were similar to those generated in the presence of the commercial kinetic inhibitor, PVP (Fig. 4H). This skeletal growth is believed to occur as a result of the more efficient dissipation of the heat of crystallization on the crystal edges, as opposed to the interior planes, as has been previously described [16]. At higher driving forces (4.4 and 5.9°C subcooling) we demonstrated that these faces were the slowest growing regions of the crystal (Fig. 4I). Since GFP alone did not adsorb to the THF hydrate (Figs. 2–4), we postulate that the inhibition of hydrate growth by AFPs is mediated by the structure of the proteins, and these become adsorbed into the crystal below the equilibrium growth point. Previously, another control protein, cytochrome C, was not seen to effect THF hydrate growth [15]. Curiously perhaps, the plant LpAFP-GFP with low TH activity towards ice but with a higher ice recrystallization inhibition activity than some other AFPs [20], showed 1.6 fold more absorption onto polycrystalline THF hydrate (at least at the higher concentrations) than the moderately ice-active Type III AFP-GFP. However, Type III without the GFP tag appeared to have superior hydrate-shaping, but its hydrate-binding affinity could not be quantified due its vulnerability to THF denaturation at higher concentrations.

These experiments were all conducted with the model THF hydrate, and thus we thought it important to determine if these recombinant proteins could also show inhibition toward natural gas hydrates. Although some fish AFPs and insect AFPs have shown activity as hydrate inhibitors in propane hydrate [21], the GFP fusions used here have never been tested, nor have the tested hydrates been formed using a gas mixture that would be found in a high-pressure oil and gas pipeline [22]. Conditions in pipelines can vary, but deep sea lines are generally at 4°C and ≥1 000 PSI (6.9×10^4^ mbar) [7], an environment that we approximated here. These parameters did not seem to have deleterious effects on the proteins because even when the gas hydrates were thawed and the proteins used a second time, inhibition activity was still observed (not shown). All of the investigated AFPs showed hydrate inhibition as determined by gas uptake assessments. Similar to the observations on the single THF crystals, LpAFP-GFP and Type III AFP-GFP showed hydrate inhibition that was modestly higher than the chemical inhibitor PVP. GFP showed little inhibition, possibly only due to a colligative effect since, as indicated, no incorporation into THF hydrate was seen. Of the additives tested, Type III AFP was demonstrably superior with an overall 18% decrease in gas hydrate formation, validating again the observations made with this protein on THF single hydrate crystals.

In conclusion, we have demonstrated for the first time that AFPs irreversibly adsorb to sII hydrate surfaces and we speculate that they act as inhibitors by binding to the {111} faces of the these symmetrical cubic crystals. We further consider that the identified ice-binding residues of these proteins may not be identical to the residues that interact with the hydrate surface, but the way is now clear for such an investigation. In addition, these experiments have established that AFPs are suitable models for understanding hydrate-inhibitor reactions and offer the prospect that these proteins, or their modified cognates, will be useful as new and more effective biologically-based hydrate inhibitors.

## Materials and Methods

### Bacterial Strains and THF Solutions

Sequences encoding the AFPs were expressed in *E. coli* BL21 cells. The plasmids used included the pET-24 vector (Novagen) for the expression of Type III AFP (7 kDa) and LpAFP-GFP (41 kDa), and pET-20b (Novagen) for Type III AFP-GFP (32 kDa). A plasmid encoding a control GFP (25 kDa) without an AFP sequence, was made by the amplification of GFP from the pET-20 vector expressing the Type III AFP-GFP sequence using primers with a 5′-NdeI site and a 3′-HindIII site. The amplified GFP was then inserted into a pET24a vector between its NdeI/HindIII sites, followed by subsequent transformation of BL21 cells. The insert was verified by sequencing. All of the recombinant proteins were tagged with a poly(His)_6_ sequence located on the C-terminal of the expressed proteins to facilitate purification.

THF (≥99.5%, Sigma-Aldrich, St. Louis, MO, USA) was mixed with distilled water or water containing solutions of proteins or other additives, at a 1∶15 molar ratio or 1∶ 3.34 (v/v) as previously described [3]. The observed crystallization temperature was the same as published values (≤4.4°C) [15].

### Bioreactor: Production and Purification

Recombinant *E. coli* BL21 cells were grown in a New Brunswick Bio-Flow 110 bioreactor (Edison, NJ, USA) using Luria-Bertani (LB) media enriched with 5 g/L yeast extract, 6 g/L glucose, 12 g/L Na_2_HPO_4_, 6 g/L K_2_HPO_4_, 2 g/L NH_4_Cl, 0.022 g/L CaCl_2_ and 0.482 g/L MgSO_4_ at 37°C, pH 7, 650 RPM agitation and 4 L/min air flow, until the culture reached OD_600_ = 8 or the DO levels were at a minimum (0–20%). The culture temperature was then decreased to 20°C, and recombinant protein expression was induced with 1 mM IPTG for 16 h. The cells were harvested by centrifugation (6 000×g, 4°C, for 20 min) and resuspended in cold lysis buffer (20 mM Tris, 500 mM NaCl, pH 7, containing an EDTA-free protease inhibitor cocktail (Mini pills; Roche, Manheim, Germany).

The pelleted cells were then lysed using a Branson sonicator for 4 min with 1 min cooling on ice and centrifuged (16 000×g, 4°C for 40 min) to remove solid cell debris. The supernatant was then mixed with cobalt-based agarose Talon Metal Affinity Resin (BD Bioscience, Mountain View, CA, USA) and incubated for 1.5 h at 4°C with mild shaking, allowing the poly(His)-tagged proteins to bind to the resin. The supernatant/resin mixture was then loaded onto a 100 ml column and the resin was washed twice with wash buffer (20 mM Tris, 500 mM NaCl, pH 7), once with wash buffer containing 5 mM imidazole, followed by 10 mM imidazole and finally eluted with 20 mM and 250 mM imidazole. The protein was visualized on a 12% SDS-PAGE gel stained with Coomassie Brilliant Blue R-250 to assess purity. Protein concentration was determined using dye-binding via bicinchurinic acid assay (BCA Protein Assay Kit; Pierce, Rockford, IL, USA). Thermal hysteresis (TH) was measured as previously described [23] with a nanolitre osmometer at protein concentrations of approximately 4 mg/ml (Clifton Technical Physics, Hartford, NY, USA).

### Polycrystalline THF Hydrate

Polycrystalline THF hydrate was grown on a hollow brass finger connected to a temperature-programmable 1197P water bath (VWR International, Mississauga, ON) filled with a water/ethylene glycol mixture (at 3∶1 v/v). A solution of THF/water (1∶3.34, v/v; 80 ml) was cooled to 4°C and poured into a 100 ml glass beaker, placed in an insulated box. The cooled brass finger (2.5°C) was submerged into the THF/water solution, which was stirred with a magnetic stir bar. To expedite hydrate formation, a small piece of THF hydrate crystal was added into the solution, which helped nucleate a thin layer of hydrate on the surface of the brass finger, a process known as seeding. When the THF hydrate uniformly covered the finger, the beaker was removed and replaced with another containing 80 ml of the test solution, and sealed with parafilm to prevent the evaporation of THF.

Triplicate test solutions of consisted of THF/water (1∶3.34, v/v; 80 ml) containing 2 µM, 4 µM, 8 µM and 16 µM of purified recombinant LpAFP-GFP, Type III AFP-GFP and GFP. THF polycrystals were grown for 8 h with the ethylene/glycol bath set to an initial temperature of 0°C and a final temperature of −6.5°C, with a 0.5°C drop every 30 min. The temperature of the THF/water/protein solution surrounding the growing crystal remained at approximately 4.9°C for the duration of the experiment. At the conclusion of the growth period, the beaker was removed and the adhered polycrystal was wrapped in aluminum foil and placed in ice to prevent melting. The bath temperature was then increased to 5.5°C, heating the brass finger and allowing the crystal to be collected.

After washing the crystal with 30 ml of distilled water (<4°C) to remove any residual solution, it was weighed and examined under UV light (midpoint wavelength  = 302 nm) at 4°C and photographed. After melting the polycrystals at room temperature, the solution was concentrated to 2 ml with a 15 ml concentrating tube (Millipore, Billevica, MA, USA), and the protein concentration was determined as above. The amount of recombinant protein adsorbed per gram of hydrate crystal (denoted as mol/g_crystal_) was calculated and a Tukey honestly significant difference (HSD) test was used for statistical comparisons (α = 0.05, q* = 3.07).

### Single Crystal THF Hydrate

Single THF hydrate crystals were formed at 3°C (1.4°C supercooling) by placing a glass Pasteur pipette, held in place by a central hole punched through a rubber stopper, into a beaker sealed with parafilm containing 80 ml THF solution. Recombinant AFPs, GFP or a commercial hydrate inhibitor, PVP (MW 10 000; Sigma-Aldrich, St. Louis, MO, USA) were added at 15 µg/ml (corresponding to 2.2 µM Type III AFP, 0.47 µM Type III AFP-GFP, 0.37 µM LpAFP-GFP, 0.60 µM GFP and 1.5 µM PVP from here on in), 50 µg/ml (corresponding to molar concentrations of 7.4 µM, 1.6 µM, 1.2 µM, 2.0 µM and 5.0 µM), 100 µg/ml (corresponding to molar concentrations of 15.0 µM, 3.1 µM, 2.4 µM, 4.0 µM and 10.0 µM) and 200 µg/ml (corresponding to molar concentrations of 30.0 µM, 6.3 µM, 4.9 µM, 8.0 µM and 20.0 µM) to observe changes in THF single crystal morphologies. The experiments were performed using wt/vol as opposed to molar concentrations because previous experiments using this technique were done in a similar fashion [16], [17]. In contrast to earlier studies [14], [17], single crystals were never transferred to inhibitor solutions, which can result in additional nucleation sites. To determine the effects of high supercooling rates on crystal structure, some crystals were grown at 0°C and −1.5°C. Crystal growth was initiated inside the pipette by nucleating the solution with a supercooled copper wire placed in dry ice, as described [17]. THF hydrate was formed down the pipette's decreasing diameter until a single, octahedral crystal emerged from the tip. If more than one crystal was initiated, or the beaker was jarred, the experiment was discontinued, as this resulted in polycrystalline growth. The crystal was grown slowly for approximately 6 h, or until the crystal edges touched the beaker walls. When complete, the crystal was removed from the solution and frozen at −20°C until photographed (Nikon, Coolpix S10).

### sII Gas Hydrate

Recombinant proteins Type III AFP-GFP, LpAFP-GFP, Type III AFP and GFP, as well as PVP were mixed at 0.1 mM concentrations with 1 000 Å pore diameter silica gel (Silicycle Chemicals, St. Jean-Baptiste, QC) at a ratio of 1 to 1.3 (v/w) respectively. The inclusion of the gel allowed more predictable and consistent nucleation times [24]. It is composed of an organic form of silicon and not amorphous silica, to which other AFPs have been reported to adsorb [25]. Briefly, the silica sand/additive mix was added into a stainless steel cell. After the sample system was immersed into a water bath set to 25°C, the sample was purged twice by a natural gas mix consisting of 2% propane, 5% ethane and 93% methane (Linde Canada, Mississauga, ON) at approximately 250 PSI (1.7×10^4^ mbar), and then was pressurized to 1000 PSI (6.9×10^4^ mbar) with the same gas mixture. Hydrate nucleation was initiated by transferring the pressurized cell into another water bath set to 0.5°C. Hydrate formation was monitored by a sudden drop in pressure (recorded using an Omega DAQPRO-5300; Fourier Systems, Fairfield, CT, USA). Moles of gas consumed (nGas), indicating the amount of hydrate formed, were calculated as previously described [26] with a minor modification.
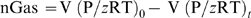



This relationship allows the determination of the difference between moles of gas at time *t* = 0 and the number of moles of gas at time *t*, where the V is the volume of the gas phase, or the cell volume minus the sample volume, P is pressure, R is the gas constant, T is temperature and *z* is the compressibility factor, calculated by Pitzer's correlations [27] and assuming no change in gas phase volume coincident with hydrate formation. Additionally, the rate of change of gas consumption, denoted as nGas/min (indicative of the rate of hydrate growth), was calculated by determining the average of the slopes of all nGas data points (slope  =  ΔnGas/Δtime). Experiments were performed in duplicate.
